# Analysis of Primary Field Shielding Stability for the Weak Coupling Coil Designs

**DOI:** 10.3390/s20020519

**Published:** 2020-01-17

**Authors:** Jiangbo Huang, Haowen Wang, Zhihong Fu, Wei Fu

**Affiliations:** 1 School of Electrical Engineering, Chongqing University, Chongqing 400044, China; 19990002@yznu.edu.cn (J.H.);; 2 School of Robot Engineering, Yangtze Normal University, Chongqing 408100, China; 3 Operation and maintenance department, State Grid Chongqing Electric Power Company, Chongqing 400010, China; weifuhelen@outlook.com

**Keywords:** weak coupling, TEM, primary field leakage, stability

## Abstract

As an electromagnetic field conversion tool in the transient electromagnetic method (TEM), the weak coupling coils reduce the mutual inductance of its transmitter and receiver coils by special structural optimization, so the detection signal can be protruded from the primary field interference generated by the transmitter coil; thus, this kind of coil design can significantly improve the signal-to-noise ratio. However, with the popularity of drag or aerial TEM exploration, the structural stability problem caused by bumps or windage leads to non-negligible primary field leakages, thereby reducing the detection reliability. This paper incorporates the primary field shielding stability as a key indicator of the weak coupling designs and proposes a calibration scheme for this stability assessment, based on which the shielding stability of five typical weak coupling coil designs is quantitatively compared, and the relationship between the primary field density and the shielding stability explored in this study may contribute to the selection and improvement of TEM coils.

## 1. Introduction

The transient electromagnetic method (TEM) is an effective non-intrusive geophysical method, which employs loop-source TEM devices in ground exploration [1,2,3,4,5,6,7]. Due to the mutual inductance between the transmitter (TX) coil and the receiver (RX) coil, the primary field generated by the emission current in the TX coil reduces the near-surface detection accuracy.

**Figure 1:**
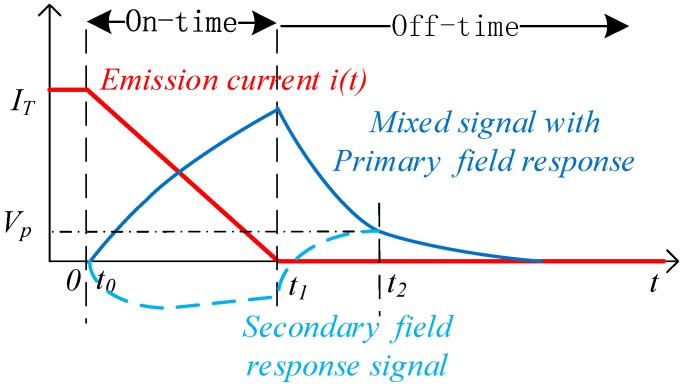
Schematic diagram of the transient electromagnetic response signal. $i_{T}\left(t\right)$ is the emission current in the TX coil; the secondary field excited by the underground anomalies is mixed with the primary field excited by the emission current in the TX coil and therefore expands the dynamic range of the signal.

**Figure 2:**
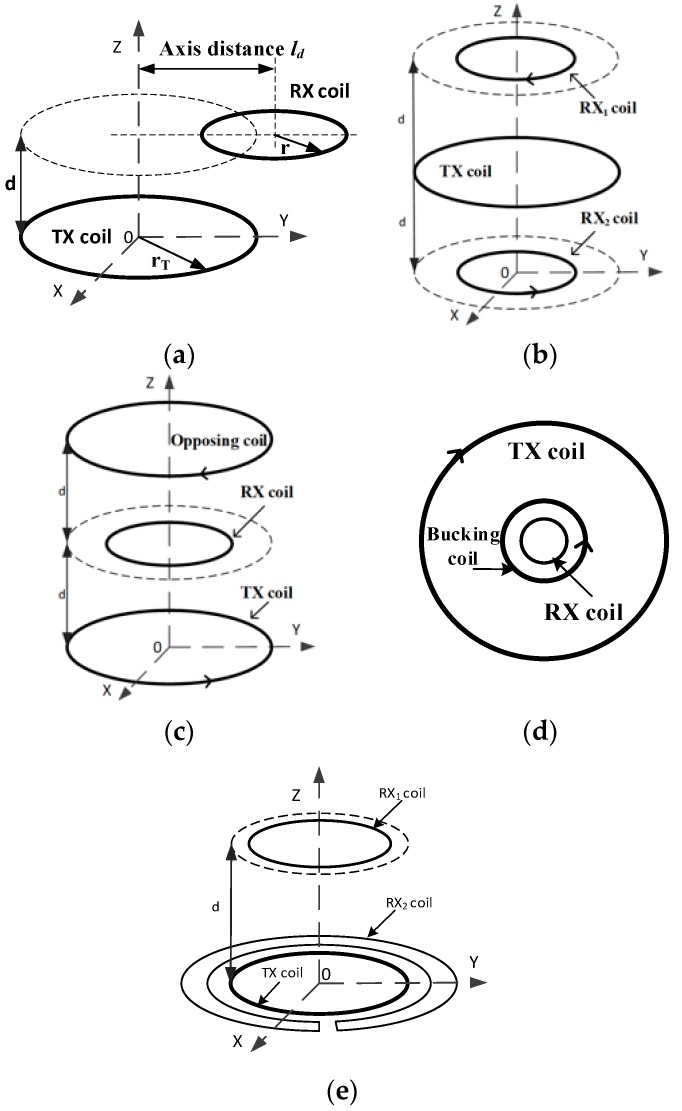
Schematic diagram of five multi-coil designs. (**a**) The eccentric coils. (**b**) The gradient design. (**c**) The opposing design. (**d**) The bucking design. (**e**) The cross-loop design.

It is very challenging to alleviate the adverse effect of the primary field on the TEM exploration from the detected signal [8,9]. TEM devices with integrated TX and RX coils in one bracket have become gradually popular in the air borne TEM detection [7] and the drag system [10]. In these devices, the relative coil locations can be set to reduce the effect of mutual inductance, thereby a pure secondary field response produced by the underground eddy current can be extracted, as shown by Figure 1. These coil arrangements are called the weak coupling coil designs, such as the cross-loop design proposed by [11], the eccentric coils used by the SkyTEM, the opposing coils proposed by [12], the gradient coils [13], and the bucking design [14]. The eccentric coils, as shown in Figure 2a, have an RX coil arranged at the edge of the TX coil, with a vertical distance $l_{d}$ and a horizontal distance d. The gradient coils and the opposing coils use two reverse connected sub-coils as the receiver or the transmitter modules, as shown by Figure 2b,c, respectively; The bucking design and the cross-loop design shown in Figure 2d,e consist of non-uniform sub-coils. Different from the reverse connected coils of the design shown by Figure 2b–d, the two sub RX coils of the cross-loop design are forward connected, so that the magnetic flux through the two sub-coils has the same polarity and no negative feedback occurs in each coil. Therefore, the cross-loop design neither suppresses the transmission magnetic moment like the opposing coils design, nor loses the secondary field flux like the gradient or the bucking coils design; thus, it has obvious advantages in detection sensitivity [11].

In theory, the weak coupling coil designs can theoretically remove the primary field response from the detection signal by adjusting the relative positions of the TX and RX coils. However, an absolutely stable shielding effect is incredible for any actual weak coupling coil. Considering the tolerances in the fabrication and installation of the coil frame, it is not guaranteed that the location of one coil will exactly obey its predicted design. Even if the tolerance can be ignored, the structural deformation caused by the electromagnetic force or the wind (in the aerial TEM) will deteriorate the primary field shielding. Therefore, the primary field shielding stability should be considered as an important indicator of the weak coupling coil designs [7].

This paper reveals the stability of the weak coupling coil designs on the primary field shielding and quantitatively compares the shielding stability of five popular coil designs, thereby providing solutions for drag or aerial TEM exploration.

## 2. The Primary Field Shielding Leakage

Here, we take the cross-loop design as an example to show the primary field leakage caused by coil offset. As shown by Figure 2e, the sub RX coil, having a smaller radius than the TX coil, is called the inner receiver coil (RX_1_ coil), while the other, having a larger radius, is called the outer receiver coil (RX_2_ coil). The RX_2_ coil is in the form of an uneven double “C” connection. These RX coils have the same winding directions. The output of the RX_1_ coil is connected in series with the input of the RX_2_ coil. Since the receiver coils RX_1_ and RX_2_ are connected across the TX coil, this structure is called the cross-loop design. In this study, the radius of a 10 turn TX coil was set as $r_{T}=0.6\;m$, the RX_1_ coil had $r_{1}=0.3\;m$ and 45 turns, while the RX_2_ coil had $r_{2}=0.65\;m$, $r_{3}=0.7\;m$, and 33 turns.

**Figure 3:**
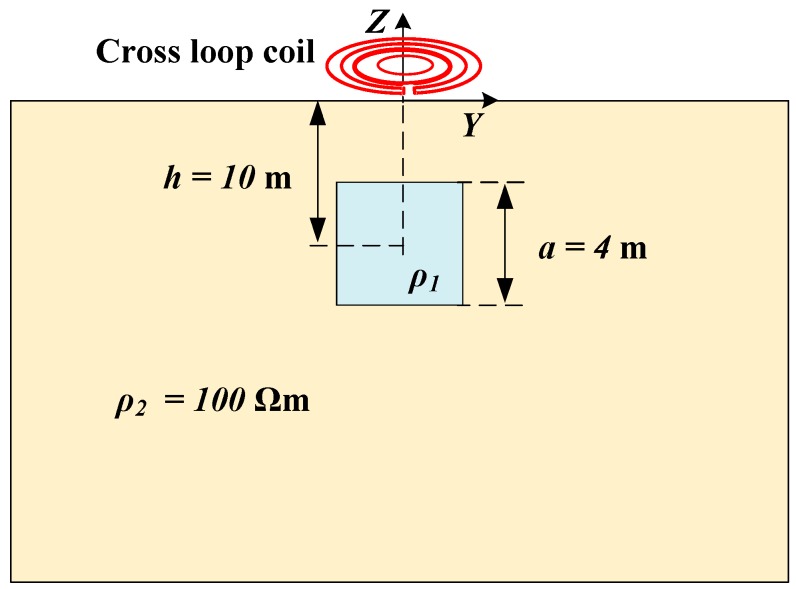
A simulation model for a resistivity anomaly. The terminal of each coil design is placed on the ground. A conductive cube is placed at h=10 m below the surface in a uniform half-space with resistivity $\rho_{2}=100\;\Omega m$. The resistivity of the cube can be either $\rho_{1}=100\;\Omega m$ or $\rho_{1}\neq 100\;\Omega m$.

A detection model for an anomalous body is depicted in Figure 3, where a conductive cube is placed at a depth of h=10 m in a uniform half-space with resistivity $\rho_{2}=100\;\Omega m$. The side of the cube is a=4 m. In the case where the resistivity of the cube is $\rho_{1}=100\;\Omega m$, the signal collected by the coil design is a uniform half-space response $u_{b}\left(t\right)$. In the case that $\rho_{1}\neq 100\;\Omega m$, the signal $u_{c}\left(t\right)$ collected by the coil design carries information about the conductive cube. The difference $u_{f}\left(t\right)=u_{c}\left(t\right)-u_{b}\left(t\right)$ is the feature signal, which exhibits the change of the uniform half-space response caused by conductive anomalies, and it is the basis of TEM for identifying the underground conductive anomalies.

**Figure 4:**
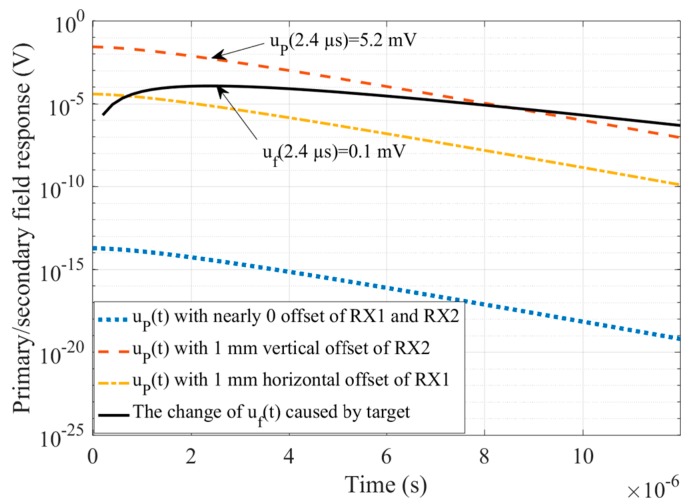
A comparison of the leaked primary field response $u_{p}$ with the effective detection signal $u_{f}$. The change of $u_{f}$ caused by a $\rho_{1}=1\;\Omega m$ cube is shown by the black solid line, and the leaked primary field response corresponding to the theoretical zero coupling state and in the case of 1 mm vertical or horizontal offset of the RX coil are respectively plotted by the blue dotted line, the red dotted line, and the yellow dotted line.

We employed ANSYS Maxwell3D electromagnetic field simulation software to calculate simulation data in the model shown in Figure 3. The ANSYS Maxwell3D electromagnetic field simulation software finds the distribution of the spatial electromagnetic field and its derivative over time based on the finite element method. It is widely used for TEM forward modeling [15,16]. For the model shown in Figure 3, the uniform half-space is emulated by a $\rho_{1}=1\;\Omega m$ cube with a side length of 120 m and the insulating boundary condition. The maximum side length of the split unit was set as 3 m. The time step of the transient magnetic field solver was 0.2 µs. The value of emission current $i_{T}\left(t\right)$ was set as $I_{T}=10\;A$ with a switch-off time $T_{off}=t_{1}-t_{0}$ as 14 μs, where $i_{T}\left(t\right)$ started to fall at $t_{0}$ and dropped to 5‰ at $t_{1}$ to take into account the exponential decay of the current tail. When the cross-loop design was in the theoretical zero coupling state, the leaked primary field response $u_{P}\left(t-t_{1}\right)$ was negligibly small, as shown by the blue dotted line in Figure 4. In the case that the RX_2_ and RX_1_ coils of the cross-loop design were vertically or horizontally offset by 1 mm, the corresponding $u_{P}\left(t-t_{1}\right)$ is shown by the red dotted line and the yellow dotted line in Figure 4, respectively. 

**Figure 5:**
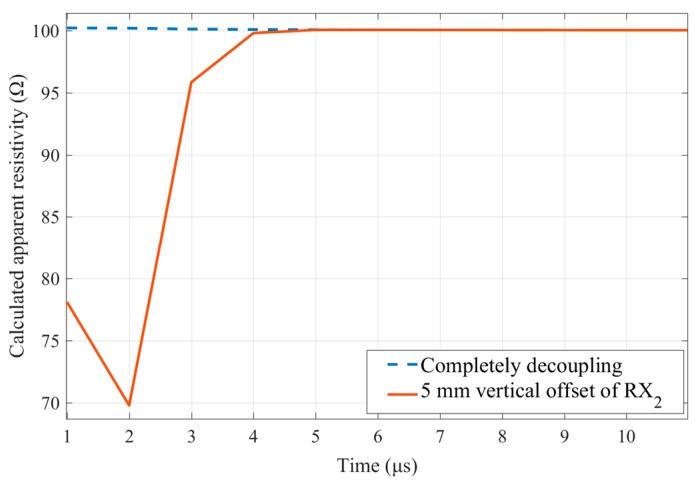
Calculation error of uniform half-space apparent resistivity caused by primary field leakage. When the RX_2_ coil is in the zero coupling position, the apparent resistivity detection result of the TEM device on the 100 Ωm uniform half-space is shown by the blue dotted line, and when the RX_2_ coil exhibits a 5 mm vertical offset, the primary field shielding leakage will lead to as high as 30% apparent resistivity calculation error, shown by the solid red line.

As can be seen from Figure 4, in the case where the RX_2_ coil was vertically offset by 1 mm, the leaked primary field response $u_{P}(t<8\;\mu s)\gg u_{f}(t<8\;\mu s)$. Especially, in the range of t≤2.4 μs, the magnitude of $u_{P}$ was at least 52 times that of the feature signal $u_{f}$. Although the leakage of the primary field response was much weaker than that of the non-weak coupling coil design, e.g., the central-loop device, the randomness of its magnitude and polarity still had an unrecoverable impact on the TEM detection data [7]. As an example, Figure 5 reveals that when the RX_2_ coil exhibited a 5 mm vertical offset, the primary field shielding leakage would lead to as high as 30% apparent resistivity calculation error for a 100 Ωm uniform half-space. Therefore, it was necessary to analyze the primary field shielding stability of the weak coupling coil designs.

## 3. Shielding Stability to the Primary Field

The conductive half-space model shown in Figure 3 is time consuming and relies on specialized algorithms or commercial software, so this section uses the conductive ring model to evaluate the shielding stability of the weak coupling coil design quantitatively.

**Figure 6:**
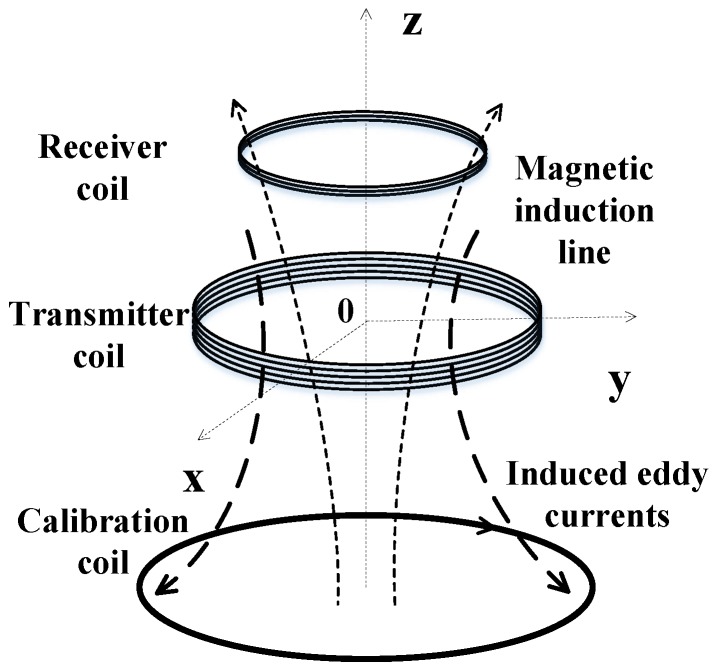
Response model based on a conductive loop. The TX coil is arranged in the *z* = 0 plane, and a conductive ring used as the secondary field source is coaxially placed in the *z* = −*h* plane below the TX coil.

The response model based on an ungrounded conductive ring is shown in Figure 6. It arranges the TX coil in the *z* = 0 plane, and a conductive ring used as the secondary field source is coaxially placed in the *z* = −*h* plane below the TX coil. Induced by $i_{T}\left(t\right)$ in the TX coil and the eddy current $i_{L}\left(t\right)$ in the conductive ring, the output signal of the RX coil can be expressed as:

(1) $$u_{t}\left(t\right)=-M_{TC}\frac{di_{T}}{dt}-M_{LC}\frac{di_{L}}{dt},$$

where $M_{TC}$ and $M_{LC}$ are the mutual inductance of the RX coil and TX coil, the RX coil, and the conductive ring, respectively. The eddy current $i_{L}\left(t\right)$ can be obtained by:

(2) $$\frac{di_{L}}{dt}+R_{L}i_{L}=-M_{TL}\frac{di_{T}}{dt},$$

where L is the inductance and $R_{L}$ the resistance of the conductive ring. $M_{TL}$ is the mutual inductance of the TX coil and the conductive ring. The pure secondary field response induced by the eddy current of the conductive ring can be obtained by:

(3) $$u_{s}\left(t\right)=-M_{LC}\frac{di_{L}}{dt}.$$

As shown in Figure 1, the primary field response was mainly distributed during the on-time period of the emission current, and it could be known from Equation (1) that the corresponding peak voltage $U_{p}$ of the RX coil could be used as an index for revealing the primary field shielding leakage. Therefore, the shielding-stability evaluation scheme is described as follows: 

When the RX coil deviates from the zero coupling position, the change of $U_{p}$ during the on-time period is recorded as 100%-α. Here, α is defined as the shielding stability coefficient, as shown in Equation (4), where $U_{p+}$ and $U_{p-}$ represent the peak voltage when the RX coil increases or decreases along the radial direction or axis of the TX coil, respectively. It is obvious that the coil design with a larger α will suffer slighter primary field leakage for a given coil offset, and thus has more satisfactory decoupling stability.

(4) $$\alpha =1-\max\left\{\frac{\left|U_{p+}-U_{p}\right|}{\left|U_{p}\right|},\;\frac{\left|U_{p-}-U_{p}\right|}{\left|U_{p}\right|}\right\}\times 100\%$$

Based on the conductive ring model shown in Figure 6, the shielding stability coefficient α of the cross-loop design was quantitatively analyzed under a preset RX coil offset; wherein the vertical stability coefficient $\alpha_{V}$ of the device was tested by presetting the position of the RX coil in the z-direction; and the horizontal stability coefficient $\alpha_{H}$ of the device was tested by adjusting the position of the RX coil along the radial direction of the TX coil.

### 3.1. Vertical Stability Coefficient $\alpha_{V}$

**Figure 7:**
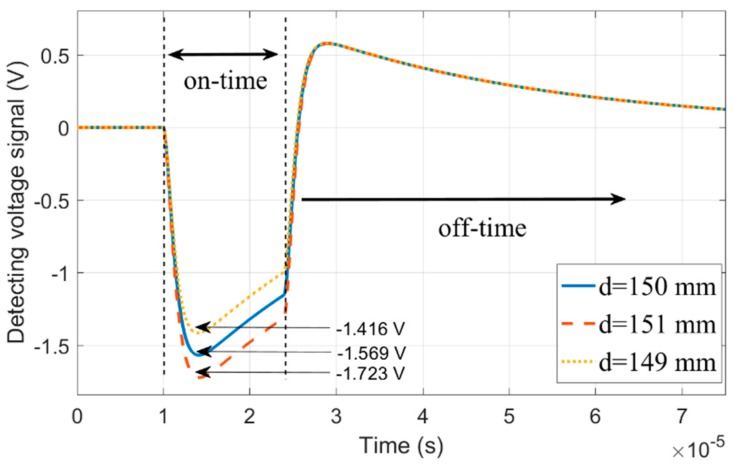
Detected signal versus time for three different *d* values. The output voltage signals of the RX coil corresponding to *d* = 150 mm, *d* = 151 mm, and *d* = 149 mm are respectively plotted by the solid blue line, the red dotted line, and the yellow dotted line, with the peak voltages $U_{p}=-1.569\;V$, $U_{p+}=-1.723\;V$, and $U_{p-}=-1.416\;V$, respectively.

In the case where the zero coupling position of the inner receiver coil RX_1_ was $r_{1}=0.3\;m$ and *d* = +150 mm, the vertical stability test result of the RX_1_ coil was as shown in Figure 7, in which the output voltage signal of the RX coil corresponding to *d* = +150 mm is marked by the solid blue line, and the voltage peak during on-time is $U_{p}=-1.569\;V$. When *d* = +151 mm and *d* = +149 mm, the corresponding output voltage waveform is shown by the red dotted line and the yellow dotted line, and its corresponding voltage peak is $U_{p+}=-1.723\;V$ and $U_{p-}=-1.416\;V$, respectively. According to Equation (4), the vertical stability coefficient was $\alpha_{V}=90.19$%, that is, while RX_1_ was shifted by 1 mm in the *z*-axis direction, the output peak voltage was shifted by 9.81% from the zero coupling status.

Similarly, the outer receiver coil RX_2_ located in the z = 0 plane had a vertical stability coefficient $\alpha_{V}$ = 98.42% for a 1 mm offset.

For the cross-loop design, the relationship between the vertical stability coefficient $\alpha_{V}$ of the RX_1_ and RX_2_ coils and the parameter *d* is shown in Table 1 and Table 2, respectively. It can be seen that the $\alpha_{V}$ of the RX_1_ coil decreased with the increase of *d*, while the $\alpha_{V}$ of the RX_2_ coil was almost stable at 98.4%. It can be seen from Table 1 and Table 2 that a smaller *d* value could effectively improve the vertical structural stability of the RX_1_ coil.

### 3.2. Horizontal Stability Coefficient $\alpha_{H}$

Taking the zero coupling position of the RX_1_ coil as *d* = 150 mm as an example, when the RX_1_ coil was coaxial with the TX coil, the voltage peak $U_{p}=-1.569\;V$. When the axis of the RX_1_ coil was offset by 5 mm from the TX coil, the voltage peak became $U_{p+}=-1.567\;V$. According to Equation (4), the horizontal stability of the RX_1_ coil was $\alpha_{H}$ = 99.81%. When the axis of the RX_2_ coil was offset by 5 mm, the corresponding $U_{p+}=-1.873\;V$, so the horizontal stability of the RX_2_ coil was $\alpha_{H}$ = 80.66%.

For the cross-loop design, the relationship between the horizontal stability coefficient $\alpha_{H}$ of the RX_1_ and RX_2_ coils and the parameter *d* is shown in Table 3 and Table 4, respectively. It can be seen that the $\alpha_{H}$ of the RX_1_ coil increased slightly with the increase of *d*, so selecting a larger *d* value could slightly improve the horizontal stability of the RX_1_ coil. The RX_2_ coil was relatively close to the TX coil, and its horizontal stability $\alpha_{H}$ was almost stable at 81%.

It can be seen from the results in Table 1, Table 2, Table 3 and Table 4 that the best decoupling stability could be obtained by placing the RX1 coil on the z = 0 plane.

## 4. Shielding Stability Comparison

Based on the conductive ring calibration model shown by Figure 6, this section compares the shielding stability coefficient of the five popular weak coupling coil designs shown in Figure 2; wherein, the vertical stability coefficient $\alpha_{V}$ of five designs was tested by presetting the position of the RX coil with an identical given offset in the z-direction; and their horizontal stability coefficient $\alpha_{H}$ was tested by adjusting an identical given offset along the radial direction of the TX coil.

We placed the bottom coil of each weak coupling coil designs on the z = 0 plane. The conductive ring with a radius of 1 m was placed coaxially 1 m below the TX coil, in which the decay constant of the eddy current was approximately 29 µs. The specific parameters are shown in Table 5. To ensure the fairness of comparison, the transmission magnetic moment and effective receiver area of each weak coupling coil designs were unified to 113 ${\mathrm{Am}}^{2}$ and 19.7 $m^{2}$, respectively.

### 4.1. The Gradient Design

Horizontal stability factor: Based on the calibration model shown in Figure 6, when both sub RX coils were coaxial with the TX coil, the peak voltage under absolute shielding was $U_{p}$ = −0.491 V, and in the case that the bottom RX coil was offset from the TX axis by 5 mm, the voltage peak $U_{p+}$ = −0.494 V, and the corresponding stability coefficient could be calculated by Equation (4) as α = 99.35%. On the other hand, when the top RX coil was offset from the TX axis by 5 mm, the peak voltage $U_{p+}$ = −0.488 V, and the corresponding stability factor α = 99.34%.

Thus, the horizontal stability coefficient of the gradient design was $\alpha_{H}$ = 99.34%.

Vertical stability factor: When the bottom RX coil was offset by +1 mm in the z-direction, the voltage peak $U_{p+}$ = −0.682 V, and α = 61.12%. When the bottom RX coil was shifted by −1 mm in the z- direction, the voltage peak $U_{p-}$ = −0.305 V, and α = 62.13%. On the other hand, when the top RX coil was offset by +1 mm in the z-direction, the voltage peak $U_{p+}$ = −0.686 V, α = 60.27%, and when the top RX coil was shifted by −1 mm in the *z*-axis direction, the voltage peak $U_{p-}$ = −0.303 V, α = 61.61%.

Therefore, the vertical stability coefficient of the gradient design was $\alpha_{V}$ = 60.27%.

### 4.2. The Opposing Design

Horizontal stability factor: Based on the calibration model shown in Figure 6, when the RX coil was coaxial with the TX coil and the opposing coil, the voltage peak $U_{p}$ = −0.54 V, and in the case that the RX coil was offset from the TX axis by 5 mm, the change in the peak voltage was negligible; thus, the horizontal stability factor could be marked as $\alpha_{H}$ = 99.99%.

Vertical stability factor: When the RX coil was offset by +1 mm in the *z*-axis direction, the voltage peak $U_{p+}$ = −0.951 V, α = 27%, and when the RX coil was shifted by −1 mm in the *z*-axis direction, the voltage peak $U_{p-}$ = −0.164 V, α = 29.75%.

Therefore, the vertical stability coefficient of the opposing design was $\alpha_{V}$ = 27%.

### 4.3. The Bucking Design

Horizontal stability factor: Based on the calibration model shown in Figure 6, when the RX coil was coaxial with the TX coil, the voltage peak $U_{p}$ = −0.961 V, and when the RX coil was offset from the TX axis by 5 mm, the voltage peak $U_{p+}$ = −1.568 V, with the stability factor α = 36.74%.

Thus, the horizontal stability coefficient of the bucking design was $\alpha_{H}$ = 36.74%.

Vertical stability factor: When the RX coil was offset by +1 mm in the *z*-axis direction, the voltage peak $U_{p+}$ = −0.912 V, α = 94.92%, and when the RX coil was shifted by −1 mm in the *z*-axis direction, the voltage peak $U_{p-}$ = −0.915 V, α = 95.22%.

Therefore, the vertical stability coefficient of the bucking design was $\alpha_{V}$ = 94.92%.

### 4.4. The Eccentric-Coils

Horizontal stability factor: Based on the calibration model shown in Figure 6, when the RX coil was in the z = +150 mm plane, its zero coupling axis distance between the TX coil was 722.66 mm, and the voltage peak $U_{p}$ = −1.067 V. When the RX coil was moved outward 5 mm away from the TX axis. the voltage peak $U_{p+}$ = −2.706 V, and the stability factor α = 53.58%. When the RX coil moved 5 mm horizontally close to the TX axis, the voltage peak $U_{p-}$ = −0.605 V, and the stability coefficient α = 56.72%.

Thus, the horizontal stability coefficient of the eccentric-coils was $\alpha_{H}$ = 53.58%.

Vertical stability factor: When the RX coil was offset by +1 mm in the *z*-axis direction, the voltage peak $U_{p+}$ = −0.917 V, and α = 85.92%. When the RX coil was shifted by −1 mm in the *z*-axis direction, the voltage peak $U_{p-}$ = −1.219 V, and α = 85.73%.

Therefore, the vertical stability coefficient of the eccentric coils was $\alpha_{V}$ = 85.73%.

**Figure 8:**
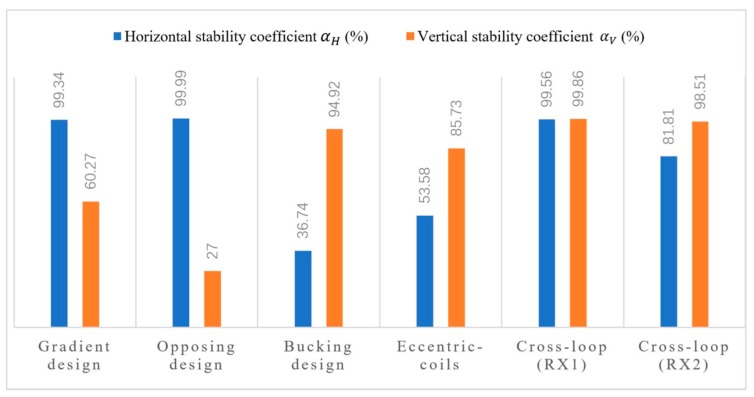
Comparison of the stability coefficients of five weak coupling coils. The horizontal stability coefficient $\alpha_{H}$ is displayed as a blue histogram, and the vertical stability coefficient $\alpha_{V}$ is displayed as an orange histogram. The best performance for the horizontal stability is the opposite design, and the bucking design is relatively disappointing; while the cross-loop design gas the best vertical stability, and the opposite design performs the worst in this respect.

The stability coefficients of the five weak coupling coil designs are compared in Figure 8. It can be seen that the cross-loop design had the best vertical stability, and the opposite design performed the worst in this respect, which required more reliable vertical rigidity to reduce the displacement perturbation in the *z*-axis. It can be seen from Figure 8 that the best performance for the horizontal stability was the opposite design, and the bucking design was relatively disappointing. The dense magnetic field distribution around the TX coil made the RX coil near it very sensitive to any displacement. Given the fact that the bucking design’s RX coil was close to the bucking coil, the high density primary field environment reduced its horizontal stability, as did the cross-loop design’s RX_2_ coil. Therefore, any RX coils of the weak coupling coil designs should be routed away from the strong primary field.

## 5. Experiment

**Figure 9:**
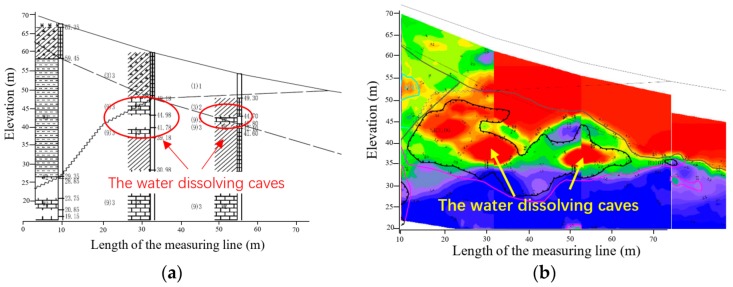
A cross-sectional view of the water dissolving cave distribution. (**a**) The exploration borehole data. (**b**) The survey result of the electromagnetic wave CT, in which the red color region represents the low resistance body and the blue color region the high resistance body.

TEM experiments were conducted to evaluate the drag investigation capability in a hill with the exploration of possible water dissolving caves. Fifteen measuring points with horizontal intervals of 5 m were evenly arranged from the summit of 65 m to the foot of 52 m above the penetration ground. Figure 9a shows the cross-sectional view of the caves from the exploration borehole data. This revealed that the anomalous bodies in the mountain were small water dissolving caves located at an elevation of 35 to 50 m. Figure 9b displays the survey results by the electromagnetic wave penetration meter. In this contour map of apparent resistivity, the red color region represents the low resistance body and the blue color region the high resistance body. This confirmed that a larger cave was located at a horizontal position of 12 to 38 m. A smaller cave located at a horizontal position of 43 to 55 m was also detected.

**Figure 10:**
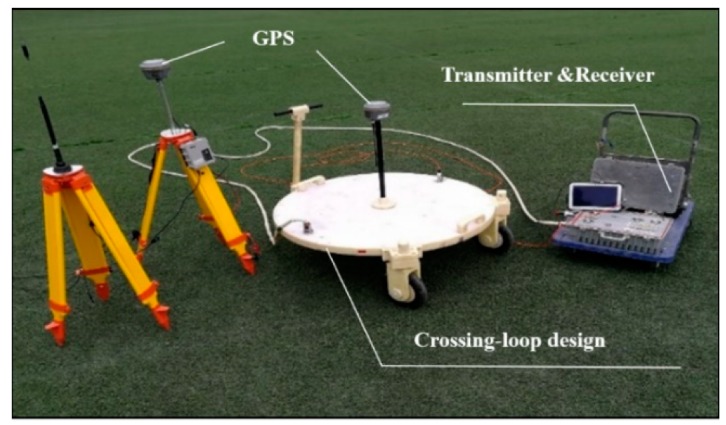
The cross-loop design and the FCTEM60 transient electromagnetic system.

**Figure 11:**
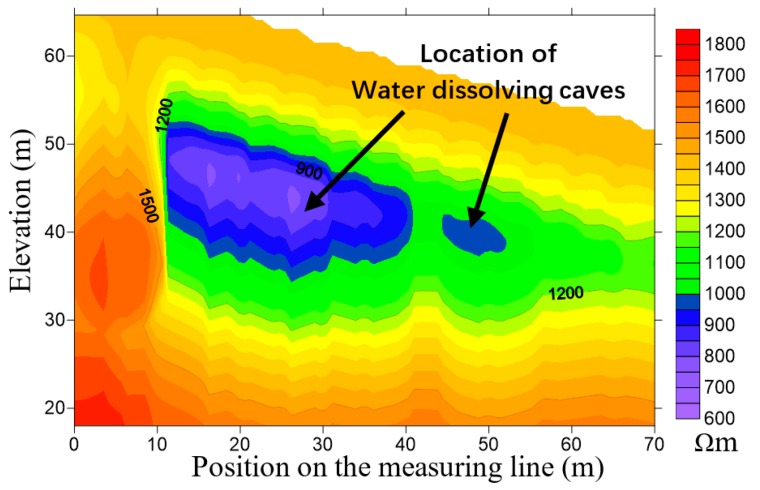
Apparent resistivity imaging of the water dissolving caves by the cross-loop design. The shape and location of the low resistance water dissolving caves are accurately displayed.

In the test, major equipment and devices included the cross-loop coils and the FCTEM60 transient electromagnetic system developed in our laboratory [17], as shown in Figure 10. The emission current was $I_{T}=65\;A$, switch-off time $T_{\mathrm{off}}=34\;\mu s$, and the effective TX area of 19.625 $m^{2}$. In this study, we employed the apparent resistivity imaging method based on the smoke ring technique to display the cross-loop design [18]. The contour map of apparent resistivity obtained by the cross-loop design is shown in Figure 11. It is observed from Figure 11 that the imaging of the water dissolving caves obtained by the cross-loop design was discernible. The exploration result was almost the same as that of the borehole electromagnetic CT. The large cave was located at the horizontal position of 12 to 35 m and a small cave located at 43~55 m in the survey line. This demonstrated that the cross-loop design was a qualified drag TEM component.

## 6. Conclusions

The shielding of the primary field response helped to improve the signal-to-noise ratio of the loop-source TEM, which popularized the application of the weak coupling coil designs. What should be paid attention to is the shielding stability of the coil structure, which tends to affect the exploration accuracy, especially in the drag or aerial TEM detection. By proposing a quantitative analysis scheme of the shielding coefficient, we compared the shielding stability factors of five popular weak coupling coil designs. The research showed that the cross-loop design had the best vertical stability, and the best performance for the horizontal stability was the opposite design; however, it required more vertical rigidity to improve the disappointing shielding stability. The worst horizontal shielding stability was found in the bucking design, which was mainly attributed to the coil arrangement. The dense magnetic field distribution around the TX coil made the nearby RX coil very sensitive to any displacement; thus, the high density primary field environment was a key factor leading to an unreliable shielding. To improve the shielding stability of the primary field response, this research suggested that any sub RX coils of the weak coupling coil designs be routed away from the strong primary field, and we hope the quantitative stability analysis of the five popular coil designs in this paper can help scholars in further research.

## Figures and Tables

**Table 1:** Comparison of the vertical stability of the inner receiver coil.

*d* (mm)	0	50	100	150	200
$\alpha_{V}$	99.86%	96.26%	92.71%	90.19%	87.94%

**Table 2:** Comparison of the vertical stability of the external receiver coil.

*d* (mm)	0	50	100	150	200
$\alpha_{V}$	98.51%	98.6%	98.37%	98.42%	98.6%

**Table 3:** Comparison of the horizontal stability of the inner receiver coil.

*d* (mm)	0	50	100	150	200
$\alpha_{H}$	99.56%	99.57%	99.67%	99.81%	99.95%

**Table 4:** Comparison of the horizontal stability of the external receiver coil.

*d* (mm)	0	50	100	150	200
$\alpha_{H}$	81.81%	80.4%	80.65%	80.66%	80.46%

**Table 5:** Parameters of the five weak coupling coil designs.

Parameter	Gradient Design	Opposing Design	Bucking Design	Eccentric-Coils	Cross-Loop Design
$I_{TX}$ (A)	10	10	10	10	10
$T_{off}\;\left(\mu s\right)$	14	14	14	14	14
*d* (m)	0.15	0.15	0	0.15	0
$r_{TX}$ (m)	0.6	0.6	0.6	0.6	0.6
$r_{RX}$ (m)	0.25	0.25	0.25	0.25	RX_1_: 0.3 RX_2_: 0.65, 0.7
